# Vitamin D supplementation and immune-related markers: an update from nutrigenetic and nutrigenomic studies

**DOI:** 10.1017/S0007114522002392

**Published:** 2022-10-28

**Authors:** Anto Cordelia Tanislaus Antony Dhanapal, Karani Santhanakrishnan Vimaleswaran

**Affiliations:** 1 Centre for Biomedical and Nutrition Research, Department of Chemical Science, Universiti Tunku Abdul Rahman, Kampar, Malaysia; 2 Hugh Sinclair Unit of Human Nutrition, Department of Food and Nutritional Sciences, University of Reading, Reading RG6 6DZ, UK; 3 The Institute for Food, Nutrition, and Health (IFNH), University of Reading, Reading, UK

**Keywords:** Vitamin D supplementation, Immunity, Gene expression, Polymorphisms, Nutrigenetics, Nutrigenomics

## Abstract

Vitamin D is both a nutrient and a neurologic hormone that plays a critical role in modulating immune responses. While low levels of vitamin D are associated with increased susceptibility to infections and immune-related disorders, vitamin D supplementation has demonstrated immunomodulatory effects that can be protective against various diseases and infections. Vitamin D receptor is expressed in immune cells that have the ability to synthesise the active vitamin D metabolite. Thus, vitamin D acts in an autocrine manner in a local immunologic milieu in fighting against infections. Nutrigenetics and nutrigenomics are the new disciplines of nutritional science that explore the interaction between nutrients and genes using distinct approaches to decipher the mechanisms by which nutrients can influence disease development. Though molecular and observational studies have proved the immunomodulatory effects of vitamin D, only very few studies have documented the molecular insights of vitamin D supplementation. Until recently, researchers have investigated only a few selected genes involved in the vitamin D metabolic pathway that may influence the response to vitamin D supplementation and possibly disease risk. This review summarises the impact of vitamin D supplementation on immune markers from nutrigenetics and nutrigenomics perspective based on evidence collected through a structured search using PubMed, EMBASE, Science Direct and Web of Science. The research gaps and shortcomings from the existing data and future research direction of vitamin D supplementation on various immune-related disorders are discussed.

One billion people worldwide are estimated to have insufficient vitamin D levels mainly due to less exposure to sunlight and poor vitamin D dietary intake. Vitamin D_3_ is a secosteroid synthesised when 7-dehydrocholesterol is exposed to UV-B via a non-enzymatic reaction^(1)^. The surge in the cases of COVID-19 with the evolution of different variants necessitates the need to treat and prevent disease escalation. Vitamin D is considered one of the inexpensive and low-risk molecules that elicit an immune-regulating response. Vitamin D obtained either endogenously or from the diet must be activated before eliciting a response^(2)^. Vitamin D_3_ exerts gene regulation after hydroxylation reaction by the enzyme 1-*α*-hydroxylase (CYP27B1) that forms 1*α*,25-dihydroxy vitamin D_3_ (1,25(OH)_2_D_3_), which is metabolically active and regulates many bodily functions, including the regulation of the immune system. The enzyme 24 hydroxylase (CYP24A1) further metabolises it to the inactive 1,24,25 OHD. The active 1,25(OH)_2_D_3_ is tightly regulated by a negative feedback mechanism to prevent excessive vitamin D signalling by inhibiting renal 1-*α*-hydroxylase and stimulating the 24-hydroxylase enzymes, thereby it helps to maintain the circulating levels of serum 25-hydroxyvitamin D (25OHD)^(3,4)^. In the intestine, it stimulates calcium reabsorption and promotes osteoblast differentiation in bone matrix. The active 1,25(OH)_2_D_3_ binds to the vitamin D receptor (*VDR*) to exert this effect, and this active hormone–*VDR* complex further dimerises with the retinoid X receptor and forms 1,25(OH)_2_D_3_-*VDR*-retinoid X receptor heterodimer that translocate to the nucleus. There it binds with vitamin D responsive elements at the promoter region and stimulate the expression of the vitamin D-responsive genes. In this way, it signals both innate (antigen and antimicrobial action) and adaptive immunity (T and B lymphocyte activity) and prevents infectious and autoimmune disorders^(5)^.

Several autoimmune diseases (multiple sclerosis; rheumatoid arthritis; type 1 diabetes; inflammatory bowel disease; systemic lupus erythematosus (SLE)) have been linked to vitamin D deficiency^(6)^ and suggested to be one of the reasons for increased vulnerability towards the recent coronavirus (COVID-19) outbreak, particularly among the elderly people^(7)^. Studies have shown that 10–25 μg of vitamin D supplementation per day offers modest protection against acute respiratory infections^(8)^, and vitamin D supplementation could play an active part in reducing the synthesis of pro-inflammatory cytokines, amplifying the expression of anti-inflammatory cytokines and increasing the expression of antioxidant genes^(9)^.

Nutrigenetics and nutrigenomics emerged as new disciplines only a few decades ago and aims to explore the nutrient–gene interactions using distinct approaches to study the mechanism through which diet can influence disease development^(10,11)^. While nutrigenetics explores the coordination between the genetic make-up of an individual in response to diet, considering the underlying genetic polymorphisms, nutrigenomics examines nutrition-responsive genome activity^(12,13)^. Though many observational studies have been reported on nutrigenetic and nutrigenomic facets of vitamin D, based on epigenome and transcriptome-wide interaction in in vitro human cell lines, to date, very few studies have documented the effect of vitamin D supplementation on gene expression and immune health in human samples.

Furthermore, vitamin D supplementation and its ramifications on immune markers have always been a subject of debate, with contradictory findings and study design flaws that impede conclusive findings. This can be attributed to poor choice of vitamin D metabolite, the dosage levels, inadequate vitamin D status, frequency of intervention and little or no impact observed with high-dose supplementation on healthy individuals. Hence, the current article aims to discuss the impact of vitamin D supplementation on immune function from a nutrigenetics and nutrigenomics perspective based on evidence from population-based randomised controlled trials and epidemiological studies. Also, the future directions of vitamin D supplementation–gene interaction on immune responses in optimising health and disease treatment are also discussed.

## Data sources and search strategies

A scrupulous literature search was done using the electronic databases including PubMed, NCBI, EMBASE, Science Direct and Web of Science databases to extract all eligible articles from inception to March 2022. The investigation was not discriminated by the date of publication. Available clinical studies published in English that utilised human participants and examined the effect of interaction between vitamin D-related gene polymorphisms, vitamin D supplementation on immune-related outcomes (i.e. nutrigenetics) and the impact of vitamin D supplementation on the expression of immune-related genes were included in this review. There was no restriction on gender, age, ethnicity and study settings. All data were extracted and organised into tables that featured study design, sample population, intervention, control, duration of treatment, analysis and outcome of the study.

Search terms included variations of ‘vitamin D supplementation’, ‘immunity’, ‘inflammatory cytokines’, ‘vitamin D receptors’, ‘transcriptional regulation’, ‘adaptive immunity’, ‘innate immunity’, ‘transcriptional profiling’, ‘*VDR* gene polymorphisms’ combined with either ‘gene expression’ or ‘gene–diet interaction’. All the relevant keywords (MeSH/Entree terms) for these topics were pooled individually and searched in the databases. Later the individual searches were combined using the Boolean operator ‘AND’, and the integrated search was performed.

## Summary of included studies

**Fig. 1.**
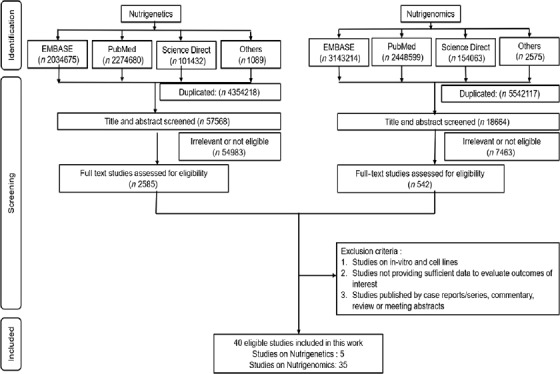
Flow diagram of the search strategy and selection of articles for the review.

A total of forty unique articles (nutrigenetics-5; nutrigenomics-35) were identified that examined the effect of vitamin D supplementation on immune-related gene expression and investigated the interactions between vitamin D genes and vitamin D supplementation on immunity (Fig. 1). PubMed search yielded twenty-four relevant articles, whereas EMBASE retrieved ten, Science Direct yielded four, while Web of Science and Medline yielded one article each. Study participants included new-borns, children, adults, elderly people and pregnant women. Both healthy volunteers and patients with diseases were part of the study population.

## Vitamin D intervention and immune response – evidence from nutrigenetic studies

Genetic epidemiological studies link molecular insights with epidemiological data that have enthused researchers in the past decade. The variations in DNA sequence that commonly occur in populations are generally termed as ‘polymorphisms’ and can provide true biological effects^(14,15)^. Their plenitude presence at the human genome and high frequencies in the human population have targeted them to explore variations in common disease risks. Although studies have reported many polymorphisms to occur in the *VDR* gene^(16)^, their influence on *VDR* protein function and signalling is still under research. Until recently, *BsmI* (rs1544410), *ApaI* (rs7975232) and *TaqI* (rs731236) polymorphisms at the 3’end of the *VDR* gene have been studied more frequently^(17–19)^. The search yielded only five nutrigenetics studies with three of the five studies reported in the Middle East population and one each from South America and Europe (online Supplementary Table 1).

Based on the evidence from Iranian population, diabetic patients (*n* 140), with *VDR FokI* (rs2228570) ff genotype, showed a lower response to circulating 25OHD levels, serum high-sensitive CRP and IL6, when supplemented with 500 ml yogurt drink (doogh) fortified with 1000 µg vitamin D_3_ for 12 weeks, and no significant changes in the serum *MMP-9, TNFα* and *IFNγ* levels^(18)^. On the other hand, women with breast cancer (*n* 28) with *TaqI* TT/Tt, *FokI* Ff genotype were more responsive to vitamin D supplementation than those with the *FokI* FF/ff and *TaqI* tt genotypes^(19)^ when supplemented with a high dose (50 000 µg of vitamin D weekly for eight weeks) and significantly increased the serum concentration of 25OHD and improved the total antioxidant capacity. Another study on breast cancer patients (*N* 214) with haplotype containing *Cdx2 G FokI f, and BsmI b* genotypes decreased the expression of *MMP9* with a low dose (4000 µg of vitamin D_3_ per day for 12 weeks) of vitamin D_3_ supplementation^(20)^.

In one study, vitamin D insufficient elderly Brazilian patients (*n* 40) with *BsmI* (rs1544410) BB/Bb genotype were more responsive to a vitamin D_3_ megadose (200 000 µg of vitamin D_3_) than the *BsmI* bb genotype and improved the circulating 25(OH)D, parathyroid hormone, ultra-sensitive-CRP and alpha-1-acid glycoprotein levels^(21)^. In the UK, a multicentre randomised controlled trial (*n* 20) on pulmonary TB and positive sputum smear patients, participants with the *TaqI* tt genotype of the *TaqI VDR* polymorphism had significantly faster sputum culture conversion and increased serum 25OHD concentrations compared with those with *FokI* genotype suggesting that those with *TaqI* tt genotype may derive clinical benefit through vitamin D supplementation^(22)^. Individual differences in response to vitamin D supplementation could be explained by epigenetic modifications and genetic diversity^(23,24)^. The ability of the individual to transform vitamin D to its active metabolites and the interaction of vitamin D_3_ with its receptors could also possibly influence individual differences to vitamin D supplementation^(25)^. Therefore, assessing the changes in epigenetic status and their respective expression of genes in immune cells may help to classify individuals as weak or strong responders to vitamin D supplementation. Unravelling the inter-relationships among gene, gene products and vitamin D are key to identifying the individuals who can be benefitted most from the vitamin D intervention strategies.

Nutrigenetic studies that probed the molecular insights of vitamin D supplementation were sparse. This creates a void in nutrigenetics research on vitamin D supplementation and immune responses with respect to study population (especially among the Asian and African nations), ethnicities, different disease states and limited longitudinal studies. This warrants for the attention of future researchers to address the missing gaps. The limitations seen were the relatively small number of SNP studied (< 30), which are not representative of a huge fraction of variation in the vitamin D pathway genes. More research is required to clearly understand the mechanism of how genetic variation and epigenetic events alter the requirements for vitamin D and their responses by expanding the metabolomics research through profiling the products of vitamin D metabolism using blood or urine samples. Also, a smaller sample size in these investigations resulted in lower study power, and a shorter supplementation duration is insufficient to conclude the outcomes of vitamin D_3_ supplementation trials. These factors must be considered while designing clinical trials in the future.

## Vitamin D intervention and immune responses – evidence from nutrigenomic studies

Numerous studies and meta-analyses have highlighted the role of vitamin D in preventing disease risk and promoting longevity^(23,25)^. However, the beneficial outcomes of vitamin D supplementation have always been under speculation based on the reports from clinical trials that challenge the non-skeletal health benefits of vitamin D as well as the detrimental changes that accompany high-dose vitamin D supplementation on bone health. It has been hypothesised that epigenetic and individual genetic differences also interfere with response to supplementation^(15,26)^. Based on the evidence from thirty-five vitamin D supplementation trials that involved a nutrigenomics approach, 51 % of the studies were from the USA, followed by the Europe and the Middle East with 17 % each, respectively. Only 3 % of the studies were contributed each by Asia and Australia, respectively, while none were reported across African nations. This highlights the lack of diversity and shortfall in this research area especially among lower middle-income countries that calls for the attention and action by the researchers to undertake vitamin D supplementation trials.

The summary of findings is illustrated in Fig. 3 (online Supplementary Table 2 and Supplementary Fig. 1). Existing evidence is discussed under three main categories that appraise the outcomes of vitamin D supplementation on immune-related markers from a nutrigenomics perspective. These include (1) the vitamin D status (sufficiency/insufficiency /deficiency) based on the circulating levels of vitamin D_3_; (2) time and dose-dependent variation and (3) choice of vitamin D supplement (vitamin D_2_/D_3_ or combination with other food or drugs).

**Fig. 2.**
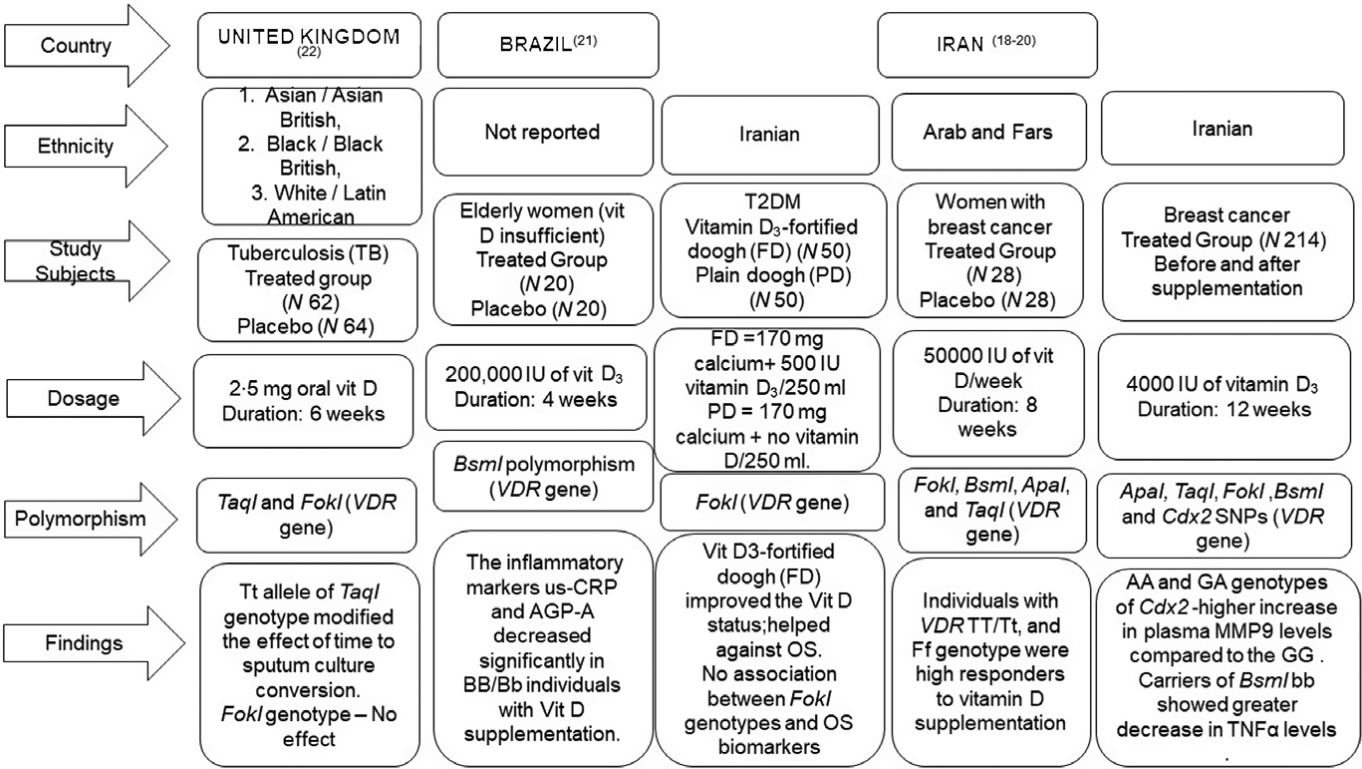
Summary of vitamin D supplementation trials and immune health from nutrigenetics perspective across different geographic terrain, ethnicities and diseased and healthy states. *VDR*, vitamin D receptor; SNP, single nucleotide polymorphism; us-CRP, ultra-sensitive C-reactive protein; AGP-A, alpha-1-acid glycoprotein; Cdx2, caudal-type homeobox 2; MMP9, matrix metalloproteinase 9; TNF*α*, tumour necrosis factor alpha; TB, tuberculosis; OS, oxidative stress; TAC, total antioxidant capacity.

**Fig. 3.**
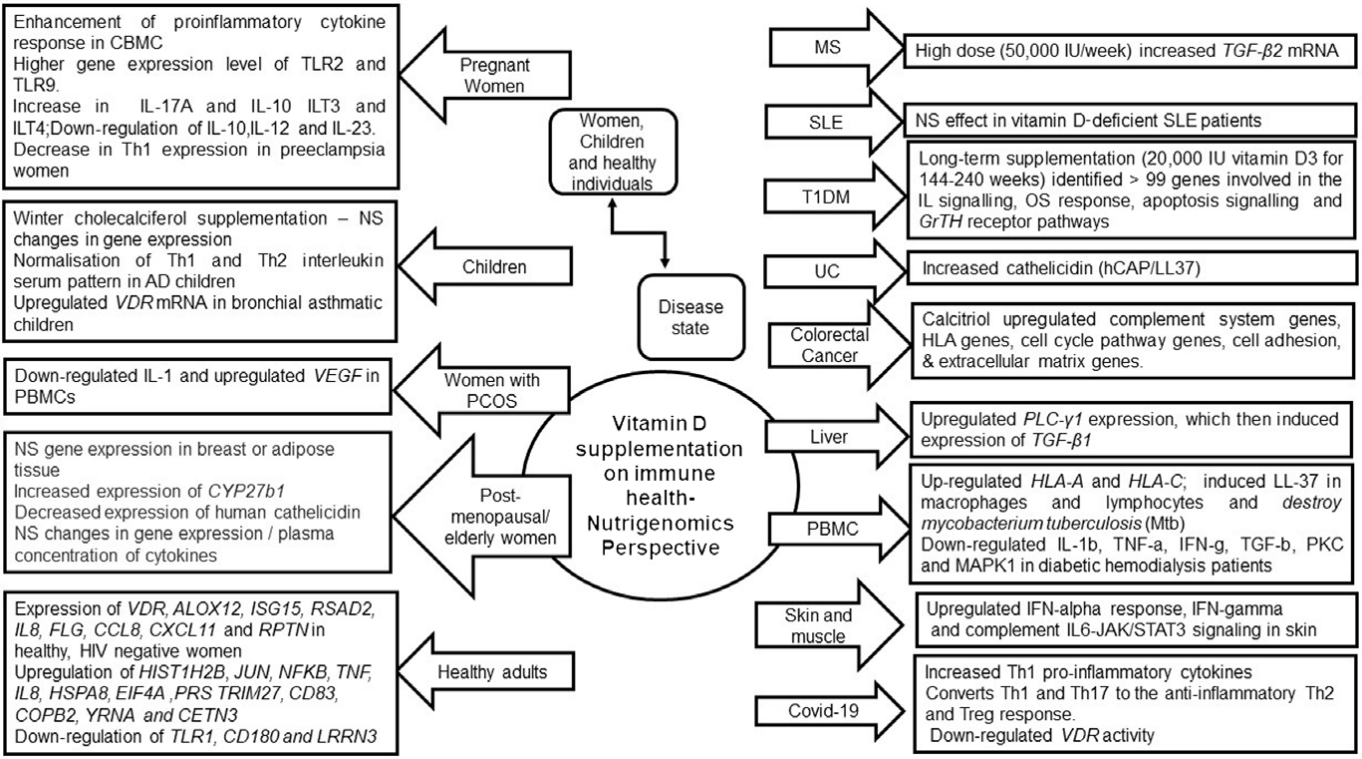
Summary of vitamin D supplementation trials and outcomes on immune health from a nutrigenomics perspective. Vitamin D_3_, cholecalciferol; NS, not significant; CBMC, cord-blood mononuclear cells; PBMC, peripheral blood mononuclear cells; TLR2, toll like receptor 2; TLR9, toll-like receptor 9; ILT3, immunoglobulin-like transcript 3; Th1, type 1 T-helper cells; Th2, type 2 T-helper cells; S100A9, S100 calcium-binding protein A9; LCN2, lipocalin-2; DEFB4, Beta Defensin 4; RSAD2, Radical S-adenosyl methionine domain containing 2; LPS, lipopolysaccharide; *VEGF,* vascular endothelial growth factor; *ALOX12,* arachidonate 12-lipoxygenase; *ISG15,* interferon-stimulated gene 15; *RSAD2*, radical S-adenosyl methionine domain containing 2; *FLG*, filaggrin; *CCL8*-C-C, motif chemokine ligand 8; *CXCL11,* C-X-C motif chemokine ligand 11; *RPTN*, repetin; *HIST1H2B,* histone H2B type 1-B; *JUN*, Jun Proto-Oncogene; *NFKB,* nuclear factor kappa B; *HSPA8*, heat shock protein family A (Hsp70) member 8; *EIF4A*, eukaryotic translation initiation factor 4A1; PRS, prieto X-linked mental retardation syndrome; *TRIM27*, tripartite motif containing 27; *CD83*, cluster of differentiation 83; *COPB2*, COPI coat complex subunit beta 2; *YRNA,* non-coding ribonucleic acids; *CETN3*, centrin 3; *LRRN3,* leucine rich repeat neuronal 3; *PLCγ1,* phospholipase C*γ*1; *TGF-β1,* transforming growth factor beta 1; *HLA–A,* human leukocyte antigen-A; *HLA–C*, human leukocyte antigen-C; *IFN*-*γ*, interferon gamma; *PKC,* protein kinase C; *MAPK1*, mitogen-activated protein kinase 1; *Mtb*, *Mycobacterium tuberculosis*.

## Influence of baseline vitamin D status on vitamin D supplementation outcomes in clinical disorders

Vitamin D deficiency impedes the health of women of all ages across the globe^(27)^. Epidemiological studies focusing on the impact of vitamin D supplementation on gene expression among women are limited to establishing the role of vitamin D on gene expression and immune health. In the PASTURE study^(28)^ (*n* 349), maternal vitamin D supplementation (Finland: 10 µg of vitamin D/day and France: a single parental dose of 2500 µg at seventh month of gestation) showed an increase in the gene expression levels of *ILT3* and *ILT4* in cord blood, which were the two hallmarks of tolerogenic dendritic cells that inhibit NF-kB activation. Similarly, in the VDAART study^(29)^, mothers supplemented with 4000 µg/d of vitamin D_3_ showed an increase in the levels of many pro-inflammatory cytokines (GM-CSF, IFN-*γ*, IL-1*β*, IL-6 and IL-8) and gene expression levels of *TLR2* and *TLR9*^(29)^ compared to the 400 µg/d group in the cord blood samples of neonates. The same study also reported that women with low vitamin D status developed preeclampsia in the early gestation stage compared with the vitamin D replete women^(30)^. These studies highlight that maternal vitamin D supplementation may induce an early tolerogenic immune response, boost the immune system of neonates and protect them from asthma-related morbidities. In addition, these studies highlight the importance of maternal vitamin D status that can influence transcriptional profiles that might contribute to fetal immune imprinting and offer protection against allergic sensitisation in early life^(31)^.

Elderly women with vitamin D insufficient status (*n* 19) administered with 50 000 µg of vitamin D/biweekly for 5 weeks failed to improve the circulating levels of hCAP *in vivo*^(21)^, while similar dosage but longer supplementation duration (3 months) in 100 vitamin D-deficient Middle Eastern women downregulated pro-inflammatory pathways by altering *TLR4/CD14* and *IFN* receptor levels and regulated NF-kB pathways that support an innate-modulated inhibition of adaptive immunity with increase in the vitamin D_3_ levels^(22)^. A pilot study in the USA on vitamin D deficient (*n* 4) and vitamin D insufficient or sufficient (*n* 4) subjects supplemented with 400 µgs (*n* 3) or 2000 µgs (*n* 5) of vitamin D_3_ daily for 2 months showed that even a slightest improvement in vitamin D status has a profound impact on the expression of genes, linked to over 160 pathways that modulate diseases associated with vitamin D deficiency^(32)^. Vitamin D deficient/insufficient SLE patients in Malta (*n* 31) supplemented with 8000 µg of vitamin D daily for 8 weeks and 8000 µg daily for 4 weeks, respectively, showed improvement in SLE disease activity^(33)^ A lower dosage (4000 µg of vitamin D_3_ daily for 12 weeks) showed contradictory result in another group of vitamin D-deficient SLE patients (*n* 19)^(2)^ where vitamin D supplementation failed to reduce the *IFN* signature. It has been postulated that to suppress the expression of the *IFN* signature-related genes and to improve disease activity, it requires higher serum 25OHD levels that remain stable for a longer time. The possible mechanism could be that vitamin D supplements may help to reduce the release of pro-inflammatory cytokines, amplify the expression of anti-inflammatory genes and enhance the gene expression involved with the antioxidant system^(34)^. It has been shown that correction of vitamin D deficiency in SLE patients suppresses the expression of genes in the interferon pathway, resulting in an improvement in SLE disease activity^(35)^. Given the evidence that correcting circulating vitamin D levels may improve disease activity in short term, future studies should address the effect of long-term vitamin D supplementation for a better understanding on the clinical and immune-related outcomes.

## Time and dose-dependent variation on vitamin D supplementation outcomes

The vitamin D requirement and optimum serum vitamin D levels to prevent disease are always a topic of scientific debate worldwide. The Institute of Medicine recommends a daily intake of 600 µg of vitamin D per day for children and adults of Canada and USA and 800 µg for older adults^(36)^. Some organisations such as the Endocrine Society recommend a daily intake of 1500 to 2000 µg to meet the optimum serum levels of vitamin D. In contrary, intakes of 10 mcg (400 µg)/d are recommended by the UK government for its citizens aged 4 years and above^(37)^. Vitamin D levels rise in response to increased vitamin D intake, in a nonlinear relationship^(38)^. However, the amount of increase depends on the baseline serum levels and supplementation period.

ODIN Junior study on healthy white children aged 4–8 years (*n* 119) reported that a high-dose winter vitamin D_3_ supplementation of 20 µg/d maintained the ability to produce calprotectin (*S100A9*) and LPS-induced IL-8 in healthy children, while low dose (10 µg/d) did not have an impact on innate immune markers^(39)^. In another study, a dose of 1000 µg/d oral cholecalciferol syrup significantly increased *VDR* mRNA expression in asthmatic Egyptian children (*n* 29)^(40)^. In addition, regular intake of vitamin D supplements among the asthmatic patients increased the serum 25OHD followed by a decrease of *VDR mRNA* expression^(40,41)^ which was also observed in cancer^(42)^ and multiple sclerosis patients^(43,44)^. The plausible reason for the increase in 25OHD followed by a reduced *VDR* expression could be that 25OHD arbitrates the binding of *VDR*-retinoid X receptor to the promoter sequence of *CYP24A1* gene that degrades 25OHD as a negative feedback mechanism^(45)^. Children with atopic dermatitis (*n* 57) administered with 1000 µg/d (25 g/d) of vitamin D for three months showed an increase in the 25OHD values and a decrease in the expression of *IL-2, IL-4, IL-6* and *IFN-γ*. This study showed that a dose of 1000 µg/d was sufficient to reduce atopic dermatitis severity in children and normalise *Th1* and *Th2* interleukin serum patterns, thus making it an effective treatment for atopic dermatitis^(46)^.

A dose of 2000 µg of vitamin D_3_/day failed to express *VDR, CYP19A1, PPARG, MCP 1* and *ADIPOQ* genes that are involved in pathways linking vitamin D status, adiposity and breast cancer risk in postmenopausal women of non-Hispanic origin^(47)^. Elderly population (Caucasians, *n* 305^(48)^; Americans, *n* 30^(49)^) supplemented with 4000 µg daily for 12 and 16 weeks, respectively, neither had an effect on gene expression (*IL-6* or *TNF*) nor on plasma concentrations of selected cytokines (*IFN-γ, IL-10, IL-8, IL-6 and TNF*)^(49)^, while contradictory result was observed in a Columbian cohort where short-term supplementation (1·5 weeks) with the same dosage increased *VDR* and *VDR* target genes (*CYP24A1* and *CAMP)* with a significant decrease in *TLR* and *CAMP* mRNA^(50)^. Meanwhile, a very high dose of 50 000 µg resulted in the expression of fifty-four differentially expressed genes in Middle Eastern women (*n* 100)^(51)^ and upregulated IFN-*α* response, IFN-*γ* response in US population^(52)^. Same dosage administered to women of Hispanic/non-Hispanic whites/non-Hispanic blacks (*n* 20) found a marked decrease in interferon-stimulated gene 15 (*ISG15*), while no significant expression was seen with *VDR, ALOX12, ISG15, RSAD2, IL8, FLG, CCL8, CXCL11, RPTN genes*^(53)^. Same dose on relapsing and remitting multiple sclerosis patients (*n* 31)^(34)^ significantly upregulated *TGF-β2 mRNA* expression in the PBMC similar to those reported in other studies^(54–56)^. Yet, some studies reported no significant effect of vitamin D supplementation in multiple sclerosis patients^(44,45,57–59)^ showing mixed results.

A high or low dose of vitamin D supplementation did not have any impact on gene expression in type 1 diabetes mellitus patients irrespective of ethnicity. Moreover, vitamin D did not defend against the decline of *β*-cell function, and neither the insulin requirement nor the metabolic control improved after diabetes onset. These findings did not support the use of vitamin D to treat adult patients with type 1 diabetes^(60–62)^. However. long-term intervention (144–240 weeks) of 20000 µg weakly to pre-diabetes patients downregulated *FPR2, CD52 IL1R2*, *GNG10* and folate *FOLR3 and* upregulated *RPS26* in a Norwegian cohort (*n* 47)^(63)^ On the other hand, ulcerative colitis patients (*n* 90) in Iran administered with 300 000 µg intramuscular vitamin D decreased ESR and high-sensitivity CRP levels and increased the expression of *LL37* supporting the beneficial role in UC patient^(64)^. A multi-centre study in Japan^(65)^ on chronic hepatitis C (CH-C) patients (*n* 18) found that cytokine IP-10 significantly decreased after 4 weeks of 1,25(OH)_2_D_3_ (1 µg/d) treatment and were able to repress the basal levels of the immune markers in the CH-C patients. This indicates that calcitriol could possibly stabilise the adaptive immune systems that were out of normal range in CH-C patients^(65)^.

In HIV-infected patients, a high-dose cholecalciferol (25 000 µg weekly) increased *CCR10* gene expression levels and reduced *CCR4* expression level of skin-homing markers, while a low dose (800 µg/d) failed to produce any immunomodulatory effects^(52)^. In contrast, 7000 µg of vitamin D_3_ significantly increased *CAMP* expression after 52 weeks and promoted antibacterial immunity in HIV-positive adolescents and young adults (*n* 48)^(66)^. Supplementing 1000 µg/d did not find any significant changes in alveolar macrophage gene expression in a study on healthy US adults (*n* 28)^(67)^, while it increased airway surface liquid antimicrobial activity and gene expression of cathelicidin antimicrobial peptide in the same population^(67)^.

The optimum dose of vitamin D supplementation to elicit a therapeutic effect has often been the subject of debate in the recent years. Vitamin D dietary guidelines suggest a minimum requirement of 600 µg/d to maximise muscle function and bone health^(68)^. However, achieving and maintaining the circulating 25OHD levels above 30 ng/ml require a minimum of 1500–2000 µg/d. Moreover, obesity and certain disease conditions demand more than the recommended dose to maintain the 25OHD levels^(68–70)^. Studies have reported that individuals with normal or near-normal levels of 25OHD, who received vitamin D supplementation above the daily requirements, did not exhibit physiological benefits^(38)^. In contrary, a high-dose vitamin D supplementation has been proved effective in many clinical conditions^(71–73)^. To have a conclusive decision, more data are required to prove that higher dose have better implications and the outcome of vitamin D supplementation depends on the baseline vitamin D levels and post-supplementation status. One of the important caveats in vitamin D supplementation is the risk that accompanies excessive vitamin D substitution, which may cause renal failure and cardiac arrest due to hypercalcemia. The upper tolerable intake limit in adults is set at 4000 µg/d (100 µg/d)^(74)^, beyond which there seems to be no additional health benefit^(37)^. Hence, while implementing high-dose vitamin D supplementation, these health impacts should also be given due consideration especially among kidney and heart patients.

## Choice of vitamin D supplementation influences expression of immune markers

Although vitamin D_2_ (ergocalciferol) and vitamin D_3_ (cholecalciferol) are structurally similar (but not identical), their functional equivalence in effecting human health has been subjected to argument in recent years with conflicting evidence reported in the literature^(75)^. A study published in 2017^(72)^ states that vitamin D_3_ increased the serum 25OHD levels compared with vitamin D_2_. In addition, the study proved that a 12-week intervention of vitamin D_2_ decreased the circulating levels of serum 1,25(OH)_2_D_3_ compared with the placebo. This decline in 1,25(OH)_2_D_3_ has been noted in several other intervention trials supplemented with vitamin D_2_^(73,76)^. Many studies have also reported the beneficial effect of vitamin D_3_ in multi-ethnic population^(71,72,77–79)^. The findings from the vitamin D_2_–D_3_ study revealed that only 13 % of down-regulated differentially expressed genes (102 of 774) were identical between the vitamin D_2_ and D_3_ treatment groups in contrast to 28 % (216 of 774) and 59 % (456 of 774) uniquely down-regulated by vitamin D_2_ and vitamin D_3_. In addition, the vitamin D_3_ supplemented group showed enhanced expression of genes involved in the interferon *α* response, which plays a critical part in combating bacterial and viral infections. Vitamin D_2_ supplemented group showed the opposite effect with no stimulatory effect of genes linked to interferon activity^(80)^. However, given that the study population was restricted to only white European ethnicity, these findings cannot be generalised.

Apart from vitamin D_2_ and D_3_, certain studies also explored the synergistic effect of vitamin D supplementation together with food or a combination of drugs on immune response. One such study (*n* 30)^(81)^ reported that co-supplementation of vitamin D and *n*-3 fatty acid (50 000 µg vitamin D/2 weeks + 2000 mg/d *n*-3 fatty acid from fish oil) significantly downregulated interleukin-1 and upregulated vascular endothelial growth factor (*VEGF)* supporting the anti-inflammatory and immunomodulatory activities of combined supplementation. Another study in a Bangladeshi population (*n* 15) found that oral vitamin D supplement (5000 µg vitamin D_3_ for eight days) co-supplemented with 500 mg phenyl butyrate produced a synergistic effect in inducing *LL-37* and caused intracellular death of *Mycobacterium tuberculosis* (Mtb) by the macrophages^(82)^. This suggests it as a potential application in treating tuberculosis. However, further studies with a larger sample size are required to confirm the antimicrobial effect of vitamin D supplementation on lung infections.

Crohn’s disease patients (*n* 9) in Denmark, supplemented with vitamin D (30 μg vitamin D_3_ daily for 1 year) along with 1200 mg Ca daily, reduced activation-induced *VDR* up-regulation in CD4 + T cells^(83)^. Another study on subjects (*n* 10) with modestly increased risk of colorectal cancer supplemented with westernised diet and 1,25(OH)_2_D_3_ (0·5 μg/d) for four weeks, significantly upregulated genes that modulate immune response and inflammation pathways^(84)^. Hence, future research can consider co-supplementation trials to further explore the synergistic effect of vitamin D with other drugs for better immunomodulatory effects.

## Nutrigenetics and nutrigenomics of vitamin D supplementation – translating evidence to practice

To date, the role of vitamin D in immune health has extended far beyond its function in bone health both in terms of basic research on gene expression as well as human trials. Though evidence has shown the effect of vitamin D on immune-related gene expression, deducing the mechanistic pathways linking vitamin D and disease is still under investigation. The current epidemiological findings can only be used to improve the vitamin D levels or correct vitamin D insufficiency. There is scarcity in quality data representing wider coverage of population, larger sample size and longitudinal studies to translate data into disease prevention and practice. This will require well-designed human trials suitable to the target population, novel and appropriate experimental approaches involving nutrigenetics, nutrigenomics and nutriepigenetics and breakthrough technologies in the vitamin D research. It is imperative to identify genetic factors that predispose the vitamin D insufficient/deficient individuals to a suboptimal vitamin D status and must be considered in future research undertakings. Investigation of the common genetic variants in vitamin D-related genes is important to distinguish population at risk for vitamin D deficiency. Furthermore, it may also enhance our understanding on heritable component of circulating vitamin D levels and its association with several diseases. Therefore, screening individuals with a high genetic risk of vitamin D deficiency may offer preventive application of personalised nutritional guidelines to foster individual vitamin D status, and thereby improve vitamin D status of the population.

## Prioritising steps to intensify research on vitamin D–gene interactions and immune health

In the milieu of limited data and competing demands to address the vitamin D–gene interactions on immune health, the evidence for the highly compelling mechanistic role of vitamin D in signalling immune system is deemed essential. Addressing the methodological challenges and uncertainties connected with vitamin D intervention should be given top priority to build sustainable nutrition solutions in the future. While developed countries are advancing towards personalised nutrition using nutritional genomics approach, it remains under explored in most of the middle-income and lower middle-income countries. Affordability to the high throughput analysis, lack of trained and skilled personnel in this newly emerged discipline, equality and ethical concerns impede research concerning this area. Cost-effective analysis, strengthening knowledge and skills in conducting nutrigenetics and nutrigenomics research, appropriate clinical designs for vitamin D intervention and wider coverage of multi-ethnic population may help to narrow down the research gaps and strengthen evidence for decision making. With over 1000 genes that are directly or indirectly regulated by 1,25OHD^(85)^, future research should henceforth focus on documenting more polymorphisms across different genes regulated by 1,25OHD to establish the functional consequences of the genetic variations. Substantial progress in this field would help to deepen our understanding of variability with respect to vitamin D endocrine system and may serve as an important health application in assessing disease risk and predicting response-to-treatment.

Another promising area is exploring the therapeutic effect of vitamin D supplementation on COVID-19 patients and documenting their immunologic response and disease outcomes. Even though observational studies support vitamin D supplementation in reducing the odds of getting respiratory tract infections, especially among vitamin D-deficient and insufficient groups, reports from randomised trials have shown mixed results that have further escalated the controversial discussion pertaining to COVID-19 and immune function. Though it is postulated that genetic susceptibility may have an impact on COVID-19 outcomes^(86)^, it is quite uncertain as to what degree these genetic factors may affect the highly affected groups despite existing genetic predisposition models and host genetic determinants^(87)^. By far, most of the vitamin D supplementation trials, both completed and ongoing in COVID-19 patients, have been used to assess the efficacy of supplementation in reducing the risk and severity of symptoms, and none of them have explored gene polymorphisms and genetic predisposition regarding COVID-19 which demand more studies in this area.

## Key findings and limitations

The findings from the scientific evidence highlight some of the key factors that need attention and action in implementing nutrigenetics and nutrigenomics approach to vitamin D supplementation in immune health. The following are the salient findings:

1. Vitamin D supplementation has demonstrated multiple immunomodulating actions, and recent studies have focussed more on establishing the physiological connection of vitamin D-mediated immunity that has direct influence on gene regulation.
2. It is evident that vitamin D supports the capability of macrophages to mature and reduces expression of inflammatory cytokines and chemokines at large that exemplifies the action of vitamin D in eliminating pathogens parallel to suppressing the potential damage caused by prolonged infection.
3. Vitamin D deficiency is widespread in the European population and North America, particularly among the elderly, which can be attributed to more than one factor, such as skin type/decreased dermal production, less sun exposure and reduced food intake rich in vitamin D_3_.
4. The immune-modulating function of genetic variants depend on the bioavailability of vitamin D. SNP can influence vitamin D levels, such as those in the *GC* gene that codes for the vitamin D-binding protein, which is linked to differential levels of circulating vitamin D. For instance, the rs7041 ‘C’ allele in *GC,* which is predominant in Caucasians, is linked to lower plasma 25OHD and elevated levels of vitamin D-binding protein in Europeans.

The limitations seen from the scientific reports are:

1. More than 50 % of nutrigenetic and nutrigenomic studies were performed among Caucasians and the whites and mainly those from the USA and other developed countries in Europe. Only a few studies were performed in Asian and African populations.
2. Lack of evidence on the replicability of vitamin D–genotype interactions on immune health in multiple ethnic groups.
3. Genetic makeup, seasonal variations, vitamin D status, physiologic and disease state, dosage levels, short-term intervention and small sample size are setbacks in drawing conclusions, and the findings cannot be extrapolated to the population at large.
4. The studies presented show mixed and controversial results that underpin the need for well-designed clinical trials to deduce the nutrigenetics and nutrigenomics aspects and strengthen data, especially in ‘omics research’ to redirect the focus on individual treatment rather than on population groups.
5. Movement control orders and the multitude of deaths due to COVID-19 halted research on COVID-19 patients and associations between vitamin D supplementation on immune markers to a greater extent.

**Fig. 4.**
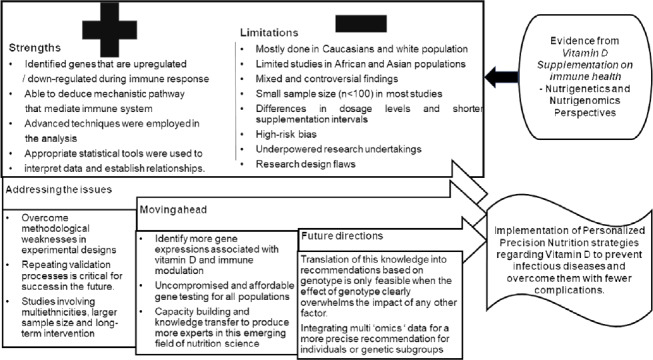
Drawing conclusions based on evidence and future directions.

However, translation of this nutrigenetics and nutrigenomics evidence into recommendations based on genotype is only feasible when the impact of genotype clearly overpowers the effect of lifestyle factors. Evidence-based research is the only stratagem that promises all data generated from nutrigenetics and nutrigenomics studies in relation to vitamin D supplementation are scrutinised before implementing personalised nutrition strategies (Fig. 4).

### Conclusion and future guidance

The evidence presented above reiterates the mechanistic role of vitamin D in regulating the immune system. Though it is highly compelling, large-scale randomised controlled trials are deemed necessary to confirm whether maintaining vitamin D sufficiency can help reduce the incidence of infections and autoimmune diseases and their severity. Studies have shown contradicting results with the ‘dose-dependent’ effect that was observed for gene expression with some results suggesting ‘the higher the dose, the more genes were affected’, while some did not show any significant gene expression with higher dosage. Further evidence from literature clearly states that even a slightest improvement in vitamin D status will have a profound impact on gene expression that execute biological functions in greater than 160 pathways associated with vitamin D deficiency. Hence, more research is required to provide definitive answer.

This review also identified novel techniques in the analysis of vitamin D signalling through next-generation sequencing technologies in primary cells, such as PBMC, that helped generate a voluminous amount of data to understand the vitamin D-triggered epigenome and transcriptome target-specific cellular systems. According to the EMBASE, PubMed, Science Direct and other databases, the US and the UK account for the highest contribution with respect to vitamin D supplementation trials on the immune response from a nutrigenetic and nutrigenomics perspectives, while it remains underexplored in Asian, African and Latin American population. The present review warrants more convincing evidence about vitamin D supplementation, gene expression and immune response, which remains uncertain. We see this as the future dimension in this area, but we are still far from recommending vitamin D for specific treatment. In conclusion, scientific and technological advances in the field of Nutrigenomics and Nutrigenetics in the state of the immune system are of prime importance to promote optimal health, which might offer greater capacity to prevent infectious diseases and overpower them with lesser complications.

## Supplementary material

For supplementary material accompanying this paper visit https://doi.org/10.1017/S0007114522002392. click here to view supplementary material
