# Vitamin D supplementation and immune-related markers: An update from nutrigenetic and nutrigenomic studies – CORRIGENDUM

**DOI:** 10.1017/S000711452200366X

**Published:** 2023-02-14

**Authors:** Anto Cordelia Tanislaus Antony Dhanapal, Karani Santhanakrishnan Vimaleswaran

**Details of correction:** The units for vitamin D have been expressed wrongly as µg instead of IU

**Corrected text should read:**

1. Pg. No:1460; Column 2; paragraph 4; line no:5 (….fortified with 1000 IU vitamin D3….) and in the same paragraph line no:10 (…with a high dose of (50 000 IU of vitamin D weekly for eight weeks)
2. Pg. No: 1461; Column 1; Paragraph 1; Line no: 2 (4000 IU of vitamin D3 per day), same column, paragraph 2; line no: 3, (200 000 IU of vitamin D3)
3. Pg. No:1462; Column 1; Paragraph 3; Line no:12 (4000 IU/d of vitamin D3 and in the same paragraph, Line no:15 (400IU/d group in the cord blood sample). On the same page and column and paragraph 4; Line no: 2 (50 000 IU of vitamin D/biweekly for 5 weeks) and line No:11 (400 IU (n 3) or 2000 IU (n 5)
  In column 2; paragraph: 1; Line no: 5 (8000 IU of vitamin D daily for 8 weeks and 8000 IU daily for 4 weeks); Line no: 7 (A lower dosage (4000 IU of vitamin D3 daily)
  Paragraph 2; Line no: 3 (a daily intake of 600 IU of vitamin D per day for children); Line no: 5 (800 IU for older adults);
  Line no: 6 to 8 (a daily intake of 1500 to 2000 IU to meet the optimum serum levels of vitamin D. In contrary, intakes of 10 mcg (400 IU)/d are recommended by the UK government)Paragraph 3; Line no: 6 (a dose of 1000 IU /d)
4. Pg. No: 1463; Column 1; Paragraph 1; Line no:1 (1000 IU/d (25mcg/d)
  Line No: 4 (1000 IU/d)
  Paragraph: 2; Line no: 1 (2000 IU of vitamin D3/d); Line no:6 (4000 IU daily for 12 and 16 weeks); Line no:14 (50 000 IU resulted)
  Column 2; Paragraph 2; Line No: 8 (20 000 IU weekly to pre-diabetes patients); Line No: 12 (300 000 IU intramuscular vitamin D)
  Paragraph 3; Line No: 1 (25 000 IU weekly)
5. Pg. No: 1464; Column 1; Paragraph 1; Line no:2 to 3 (800 IU/d failed to produce any immunomodulatory effects (53). In contrast, 7000 IU of vitamin D3)
  Line No: 6 (Supplementing 1000 IU/d)
  Paragraph 2; Line no: 4 (requirements of 600 IU/d); Line no:7 (1500-2000 IU/d)
  Column 2; Paragraph 1; Line no: 8 (4000 IU/d)
6. Page No: 1465; Column 1; paragraph 2; Line No: 5 (50 000 IU vitamin D/2 weeks); Line no:10 (5000 IU vitamin D3 for eight days)

