# Supplementary material for: Vitamin D supplementation and immune-related markers: an update from nutrigenetic and nutrigenomic studies
Source: Br J Nutr. 2022 Oct 28;128(8):1459–69. doi: 10.1017/S0007114522002392 (PMC9557210; doi:10.1017/S0007114522002392)
Supplement: Supplementary file 1 [file S0007114522002392sup.zip › S0007114522002392sup002.docx]

**Supplementary Table 1: Summary of Nutrigenetic studies focusing on the interaction between vitamin D related gene polymorphisms and vitamin D supplementation in immune health**

| S.No | Year | Country | Ethnicity | Age  (years) | Study subjects | Study Design | Assay | Vit D dosage/  Supplements | Duration | Polymorphism | Key findings | Reference |
| --- | --- | --- | --- | --- | --- | --- | --- | --- | --- | --- | --- | --- |
| 1 | 2015 | IRAN | Not reported | 30-60 | Type 2 diabetic patients | Randomized, double-blind, placebo-controlled trial /NCT01236846 | PCR-RFLP | Vitamin D3-fortified doogh (FD) group=170 mg calcium+ 500 IU vitamin D3/250 ml  Plain doogh (PD) group=170 mg calcium + no vitamin D/250 ml. | 12 weeks | *VDR FokI* variants | NS | [18](https://pubmed.ncbi.nlm.nih.gov/25424601/) |
| 2 | 2019 | IRAN | Arab and Fars | 45-50 | Women with breast cancer | Randomized, double-blind, placebo-controlled trial | SSP-PCR | Supplementation group received 50,000 IU of vitamin weekly | 8 weeks | *FokI, BsmI, ApaI, and TaqI* polymorphism | TT/Tt, Ff genotypes elevated TAC after supplementation (p<0.05). | 19 |
| 3 | 2019 | IRAN | Not Reported | 25-65 | Breast cancer survivors | Single-arm non-randomized pre- and post trial supplementation | PCR | 4000 IU of vitamin D3 daily | 12 weeks | *ApaI, TaqI, FokI and BsmI* SNPs; *Cdx2* | AA and GA genotypes of *Cdx2* showed an increase in plasma MMP9 levels  *BsmI* bb showed a greater decrease in circulating TNFα levels. | 20 |
| 4 | 2015 | BRAZIL | Not reported | >60 | Elderly women (Buccal epithelial cells) | Randomized, double-blind, placebo-controlled trial | CRP/RFLP | Supplementation group (SG): 200,000 IU of vitamin D3 and placebo group (PG) | 4 weeks | *BsmI* polymorphism of the *VDR* gene | The inflammatory markers us-CRP and AGP-A decreased significantly in BB/Bb individuals with Vit D supplementation. | 21 |
| 5 | 2011 | UK | 1. Asian / Asian British,  2. Black/ Black British,  3. White / Latin American | Median age: 30.7 | Patients with TB | Randomized, double-blind, placebo-controlled trial /NCT00419068 | RT-PCR | four oral doses of 2.5 mg vitamin D or organoleptically identical placebo | 6 weeks | *TaqI and FokI* polymorphisms of the vitamin D receptor (*VDR*) | *TaqI* genotype modified sputum culture conversion (P =0.03), *FokI* genotype NS effect. | 22 |

*VDR* – Vitamin D receptor; SNP- Single Nucleotide Polymorphism; us-CRP -Ultra-sensitive C-Reactive Protein; AGP-A – Alpha-1-Acid Glycoprotein; Cdx2- Caudal-type homeobox 2; MMP9 - Matrix metalloproteinase 9; TNFα - Tumour Necrosis Factor alpha; TB- Tuberculosis; OS -Oxidative Stress; TAC -Total Antioxidant Capacity.
