# Supplementary material for: Vitamin D supplementation and immune-related markers: an update from nutrigenetic and nutrigenomic studies
Source: Br J Nutr. 2022 Oct 28;128(8):1459–69. doi: 10.1017/S0007114522002392 (PMC9557210; doi:10.1017/S0007114522002392)
Supplement: Supplementary file 1 [file S0007114522002392sup.zip › S0007114522002392sup003.docx]

**Supplementary Table 2: Summary of Nutrigenomic studies on vitamin D supplementation in immune health**

| S.No | | Year | Country | | Ethnicity | Age  (years) | Study subjects | Study Design | Assay | Vit D dosage/Supplements | Duration | Genes studied/Key findings | Reference |
| --- | --- | --- | --- | --- | --- | --- | --- | --- | --- | --- | --- | --- | --- |
| 1 | 2019 | USA | Non-white | | 18-50 | Healthy young black and white adults | randomized controlled double-blind clinical trial | Microarray, RT-PCR | 600, 4,000 or 10,000 IU/d of vitamin D3 | 24 weeks | Upregulated *HIST1H2B, JUN, NFKB, TNF, IL8, HSPA8, EIF4A* and *PRS*  Down regulated *TLR1, CD180* and *LRRN3*. | 23 |  |
| 2 | 2010 | AUSTRIA, FINLAND, FRANCE, GERMANY, SWITZERLAND | Not reported | | Less than 30-Older than 35 years | Pregnant women and their new-borns | Prospective multi-centre cohort study [Protection against Allergy-Study in Rural Environment (PASTURE) | RT-PCR | Finland-10 mg of vit D /d.  France-a single parenteral dose of 2500 mg/d in the seventh month of pregnancy). |  | Increased ILT3 and ILT4. | 28 |  |
| 3 | 2018 | USA | African American and other races | | 18-40 | Pregnant women (2nd and 3rd trimester), cord blood mononuclear cells (CBMCs) | randomized double-blind placebo-controlled clinical trial/NCT00920621 | RT-PCR | High dose Vit D=4400 IU/d , Low dose Vit D=400 IU/d | 8 weeks | 4400 IU of vit D3 resulted in a higher gene expression level of TLR2 (P =0.02) and TLR9 (P =0.02). | 29 |  |
| 4 | 2016 | USA | Black/African American, White/Hispanic, White/non-Hispanic, Other | | <30 | Pregnant women | Randomized double blind, placebo-controlled clinical trial (nested case-control study in a subset) | RT‑PCR | Vit D treatment arm- 4,400 IU and placebo arm-400 IU | 20-22 weeks | Low vitamin D status developed pre-eclampsia in early gestation | 30 |  |
| 5 | 2016 | USA | 80% African American and 20% Caucasian | | 18–39 | Healthy first trimester women | Nested cohort study of a larger multicenter, randomized, double-blind, controlled clinical trial (NCT00920621) | NGS | Low dose (400 IU) and High dose (4400 IU) Vit D | 22 – 30 weeks | Increased GM-CSF, IFN-γ, IL-1β, IL-6, and IL-8 cytokines. Increased TLR2 and TLR9 | 31 |  |
| 6 | 2009 | USA | Not reported | | >60 | Healthy subjects with low bone mineral density | Non-randomized clinical trial | RT-PCR | 50,000 IU vitamin D2 twice weekly | 5 weeks | vit D ‘insufficient’ participants increased CYP27b but decreased human cathelicidin antimicrobial peptide hCAP mRNA. | 32 |  |
| 7 | 2013 | USA | Not reported | | 18-30 | Healthy, non-patient English speaking adults | Randomized Double-Blind Clinical Trial/NCT01696409 | RT-PCR | 400 IUs (n = 3) or 2000 IUs (n = 5) vit D3 daily | 8 weeks | Expressed TRIM27, CD83, COPB2, YRNA, and CETN3 | 33 |  |
| 8 | 2015 | USA | Black, Hispanic | | 30-40 | Patients with Systemic Lupus Erythematosus | multicenter, randomized, double-blind, placebo-controlled phase II study (NCT00710021) | RT-PCR, Micro array | 3 treatment groups = 0, 2000IU or 8000IU of vitamin D3 | 12 weeks | IFN signature response was observed in all groups | 34 |  |
| 9 | 2018 | IRAN | Not reported | | 31.25 ± 7.05 | Relapsing-remitting Multiple sclerosis (MS) patients | randomized clinical trial | RT-PCR | 50,000 IU/week | 8 weeks | Significant increase in the expression of *TGF-β2* mRNA | 35 |  |
| 10 | 2015 | IRAN | Not reported | | 30-40 | PBMCs of Patients with Multiple sclerosis | Observational study | RT‑PCR | 50,000 IU of vitamin D weekly | 8 weeks | Significant up-regulation of IL-6 and IL-17A | 36 |  |
| 11 | 2017 | DENMARK | European White | | 6.7 ± 1.5 | Healthy young kids | Randomized, placebo-controlled, double-blinded trial | RT-PCR | 0 (placebo), 10 or 20 μg/day of vitamin D3 | 20 weeks | 10 µg/day NS  20 µg/day: maintained few biomerkers | 40 |  |
| 12 | 2019 | EGYPT | Not reported | | 10-18 | Children with bronchial asthma | Observational | RT-PCR | oral vitamin D3 (cholecalciferol) syrup (1000 IU/d) | 8 weeks | Upregulated *VDR* mRNA level | 41 |  |
| 13 | 2015 | USA | Not reported | | 4 ± 3.15 years | Children with atopic dermatitis | Single-centre, prospective and longitudinal study |  | Oral vit D 31,000 IU/day | 12 weeks | Altered cytokines (IL-2, IL-4, IL-6, IFN-γ). | 47 |  |
| 14 | 2017 | USA | Non-Hispanic White  Non-Hispanic Black  Other (American Indian, Asian) | | 50–75 | overweight/  obese postmenopausal women | Double-blind, placebo-controlled randomized trial | qRT-PCR | 2000 IU/day | 48 weeks | NS effects CYP19A1, PPARG, ADIPOQ, MCP-1, and *VDR* in breast or adipose tissue. | 48 |  |
| 15 | 2018 | UK | Caucasian | | ≥65 | older people | Double-blind, placebo-controlled, randomized clinical trial | Microarray | 4000 IU / 2000 IU /Placebo | 48 weeks | NS effect on IFN-γ, IL-10, IL-8, IL-6 and TNF-α after one year of supplementation. | 49 |  |
| 16 | 2015 | USA | Not reported | | subset 1=78.5 ± 4.79, subset 2=77.9 ± 4.05 | Mobility limited women, and mobility-limited older adults | Cross-sectional, longitudinal study | RT‑PCR | 4000 IU/d | 16 weeks | Neither intramuscular IL-6 nor TNFα gene expression was observed. | 50 |  |
| 17 | 2018 | COLOMBIA | Not reported | | 18 - 50 | healthy volunteer subjects. | Exploratory study | RT-PCR | 1000 or 4000 (IU)/day | 1.5 weeks | Increased *VDR* and *VDR* target genes, specifically *CYP24A1 and CAMP*  Significant decrease in TLR and CAMP mRNA . | 51 |  |
| 18 | 2018 | IRAN | Not reported | | 18-80 | PBMCs of diabetic haemodialysis (HD) patients. | RCT | RT-PCR | 50,000 IU (n = 30) or placebo (n = 30) every 2 weeks | 12 weeks | Downregulated IL-1b , TNF-a, IFN-g, TGF-b, PKC, and MAPK1  Reduced expression of NF-kB, IL-4, IL-6, and *VEGF* | 52 |  |
| 19 | 2017 | USA | Not reported | | Oral group= 44.7 ± 12.5, UVB group= 39.2 ± 12.3 | Vit D deficient subjects | Randomized parallel-intervention study (NCT01688102) | RNA sequencing by HiSeq 2500 sequencer | vitamin D3= 50,000 IU/wk as 5 capsules of 10,000 IU.If 25(OH)D was less than 35 ng/mL, then additional dose of 50,000 IU | 8 weeks | Oral Vit D3 upregulated IFN-alpha response, IFN-gamma response in blood and complement, IFN-alpha response, IFN-gamma response, IL6- signaling in skin respectively. | 53 |  |
| 20 | 2020 | USA | Hispanic  Non-Hispanic Black  Non-Hispanic White Asian | | 21–50 | Healthy, non-pregnant, HIV-negative women | Randomized clinical trial/NCT02186535. | RT-PCR | vitamin D—4000 IU daily or 50,000 IU weekly—in capsule form and 1000 mg of calcium to take daily | 8 weeks | NS effect on *VDR, ALOX12, ISG15, RSAD2, IL8, FLG, CCL8, CXCL11, RPTN.*  Decrease interferon-stimulated gene | 54 |  |
| 21 | 2017 | NORWAY | Not reported | | 61.0 ± 8.5 | participants with prediabetes | Randomized controlled trial (RCT) | NGS, microarray | vitamin D3 (n = 47) in a weekly dose of 20,000 IU or placebo (n = 47) | 144-240 weeks | Downregulated *FPR2, CD52 IL1R2)*, *GNG10* and folate *FOLR3*  Up-regulated *RPS26* | 64 |  |
| 22 | 2015 | IRAN | Not reported | | 30-40 | Subjects with ulcerative colitis | double‑blind randomized controlled trial | qRT‑PCR | 1 mL 300,000 IU Vitamin D3 or 1 mL normal saline as placebo | 12 weeks | Significant change hCAP/LL37 Vitamin D group compared to placebo | 65 |  |
| 23 | 2013 | JAPAN | Not reported | | 29 - 71 | Chronic hepatitis-C {Genotype 1b} patients | Non-randomized clinical trial | RT-PCR | 18 Subjects received 1(OH) Vitamin D3 (1 µg/day) 24 Subjects received 1(OH) vitamin D3 (1 µg/day)/Peg-IFN/RBV 42 Case match control subjects received Peg-IFN/RBV .1 µg/day | 48 weeks | IP-10 was significantly decreased after 4 weeks. Th1 responses in the subjects treated with 1(OH) vitamin D3/Peg-IFN/RBV were significantly higher than those treated with Peg-IFN/RBV at 12 weeks | 66 |  |
| 24 | 2013 | NETHERLANDS | African, White, Hispanic | | 20-60 | PMBCs of vitamin D3-deficient HIV-infected patients | RCT | flow cytometry | Low-dose study cohort (n=9) daily oral dose of 800 IU for 3 months  High-dose cohort (n = 7) weekly oral dose of 25,000 IU for 2 months.  Control group (n = 7) no supplementation | 8-12 weeks | High-dose cholecalciferol supplementation differentially influenced skin-homing markers on Treg, increased CCR10 expression | 67 |  |
| 25 | 2014 | USA | Not reported | | 18-60 | Adults | Parallel, double-blind, placebo-controlled, randomized trial/NCT01967628. | PCR, Micro array | 1000 IU/day | 12 weeks | Marginal increase in 25-hydroxyvitamin D levels in supplemented group. | 68 |  |
| 26 | 2022 | UK | South Asian and White European | | 20-64 | Post-menopausal | A randomized, double-blind, placebo-controlled food-fortification trial | "Transcriptome analysis  Microarray " | placebo juice with placebo biscuit (placebo), juice supplemented with 15 μg vitamin D2 with placebo biscuit (D2J), placebo juice with biscuit supplemented with 15 μg vitamin D2 (D2B), juice supplemented with 15 μg vitamin D3 with placebo biscuit (D3J), or placebo juice with biscuit supplemented with 15 μg vitamin D3 (D3B). | 12 weeks | 28% (216 of 774) downregulated by D2  59% (456 of 774) downregulated by D3  D3 enhanced interferon alpha response | 81 |  |
| 27 | 2018 | IRAN | Not reported | | 18 - 40 | women with polycystic ovary syndrome (PCOS) | RCT | RT-PCR | 50,000 IU vitamin D every 2 weeks plus 2000 mg/day omega-3 fatty acid from fish oil (n=30) or placebo (n=30) | 12 weeks | Downregulated IL-1 and upregulated *VEGF* | 82 |  |
| 28 | 2013 | BANGLADESH | Not reported | | 18 - 55 | PBMCs of Healthy volunteers | Non-clinical open trial/NCT01580007 | RT-PCR | 5000 IU vitamin D3 for eight days | 40 days | Induced LL-37 and caused intracellular death of Mycobacterium tuberculosis (Mtb) by the macrophages | 83 |  |
| 29 | 2015 | DENMARK | Not reported | | 20-60 | PBMCs of patients with Crohn’s disease | randomized placebo-controlled clinical study/NCT 0013 2184 | RT‑PCR | 30 µg vitamin D3 or placebo daily. All patients received 1200 mg calcium daily | 480 weeks | *VDR* up-regulation was inhibited by 30% in vit D treatment group compared to placebo | 84 |  |
| 30 | 2016 | USA | African American and White | | 48-72 | Healthy subjects at modestly increased risk of colorectal neoplasia with a | Randomized crossover clinical trial/ NCT00298545 | Microarray | Study A:1. Western Diet (WD) supplemented with and without 2 g calcium carbonate/d and 2) a 4-wk WD supplemented with 1,25(OH)2D3 (0.5 mg/d) with or without 2 g calcium carbonate | 4 weeks | WD upregulated immune and/or inflammatory pathways but significantly downregulated the nuclear factor of activated T cells pathway. | 85 |  |

VitD3 – Cholecalciferol; NS – Not significant; CBMC – Cord- blood mononuclear cells; PBMC - Peripheral blood mononuclear cells; TLR2- Toll like receptor 2; TLR9 – Toll like receptor 9; IL-17A- Interleukin 17A; IL-10 -Interleukin 10; ILT3 - Immunoglobulin-like transcript 3; Th1- Type 1 T helper cells; Th2- Type 2 T-helper cells; S100A9 - S100 calcium binding protein A9;LCN2 – Lipocalin-2; DEFB4- Beta Defensin 4; RSAD2- Radical S-Adenosyl Methionine Domain Containing 2; LPS – Lipopolysaccharide; CYP27b1 - *VEGF-* Vascular endothelial growth factor; *ALOX12*- Arachidonate 12-Lipoxygenase; *ISG15-*Interferon-stimulated gene 15; *RSAD2*-Radical S-Adenosyl Methionine Domain Containing 2; *FLG* -Filaggrin; *CCL8*-C-C Motif Chemokine Ligand 8; *CXCL11* -C-X-C Motif Chemokine Ligand 11; *RPTN* -Repetin; *HIST1H2B* -Histone H2B type 1-B; *JUN* -Jun Proto-Oncogene; *NFKB* -Nuclear factor kappa B; *HSPA8* -Heat Shock Protein Family A (Hsp70) Member 8; *EIF4A* -Eukaryotic Translation Initiation Factor 4A1; PRS - Prieto X-Linked Mental Retardation Syndrome; *TRIM27*-Tripartite Motif Containing 27; *CD83* -Cluster of Differentiation 83; *COPB2* -COPI Coat Complex Subunit Beta 2; *YRNA* – non-coding ribonucleic acids; *CETN3* - centrin 3; *LRRN3* -Leucine Rich Repeat Neuronal 3; *PLCγ1* -Phospholipase Cγ1; *TGF-β1*- Transforming growth factor beta 1; *HLA -A* -Human leukocyte antigen-A; *HLA -C* -Human leukocyte antigen-C; *IL-1β* -Interleukin 1 beta ; *IFN*-γ - Interferon gamma; *PKC* - Protein kinase C; *MAPK1* -Mitogen-Activated Protein Kinase 1; *Mtb* -*Mycobacterium tuberculosis*
