# Supplementary figures and images for: Vitamin D supplementation and immune-related markers: an update from nutrigenetic and nutrigenomic studies
Source: Br J Nutr. 2022 Oct 28;128(8):1459–69. doi: 10.1017/S0007114522002392 (PMC9557210; doi:10.1017/S0007114522002392)

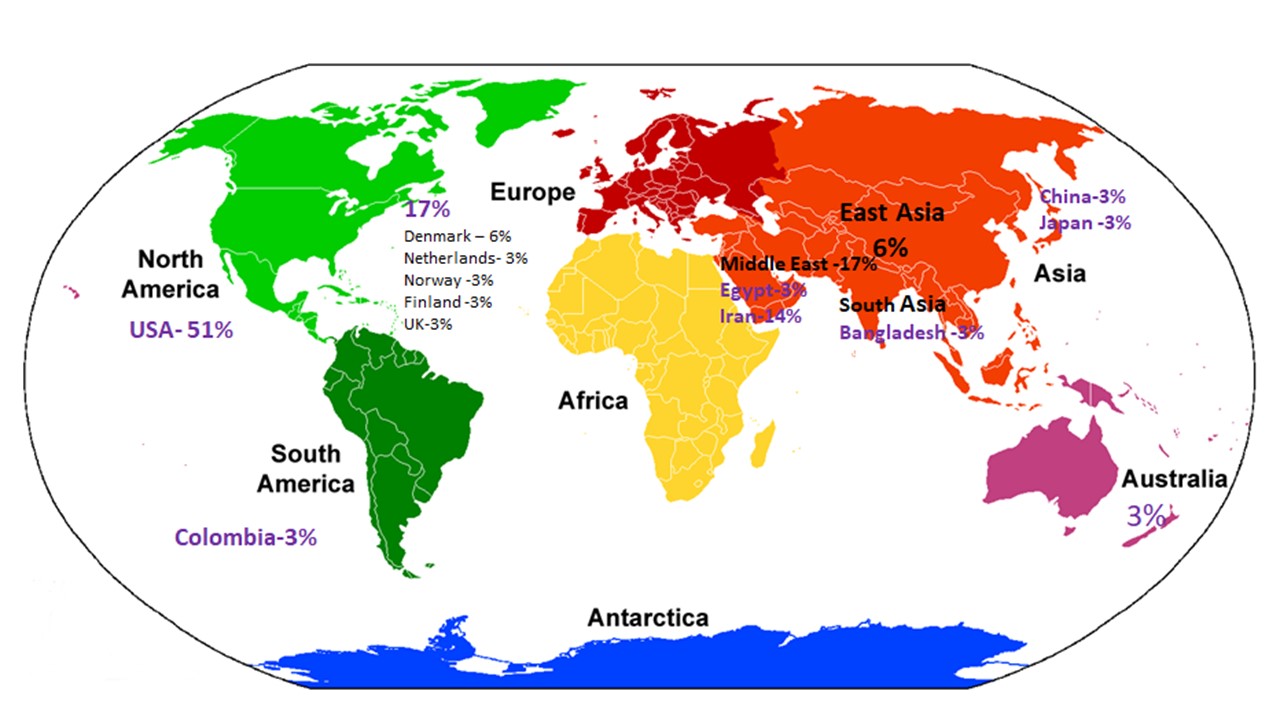

Supplement: Supplementary file 1 [file S0007114522002392sup.zip › S0007114522002392sup001.jpg]
